# Using Random Walks to Generate Associations between Objects

**DOI:** 10.1371/journal.pone.0104813

**Published:** 2014-08-25

**Authors:** Muhammed A. Yildirim, Michele Coscia

**Affiliations:** Center for International Development, Harvard University, Cambridge, Massachusetts, United States of America; University of East Piedmont, Italy

## Abstract

Measuring similarities between objects based on their attributes has been an important problem in many disciplines. Object-attribute associations can be depicted as links on a bipartite graph. A similarity measure can be thought as a unipartite projection of this bipartite graph. The most widely used bipartite projection techniques make assumptions that are not often fulfilled in real life systems, or have the focus on the bipartite connections more than on the unipartite connections. Here, we define a new similarity measure that utilizes a practical procedure to extract unipartite graphs without making *a priori* assumptions about underlying distributions. Our similarity measure captures the relatedness between two objects via the likelihood of a random walker passing through these nodes sequentially on the bipartite graph. An important aspect of the method is that it is robust to heterogeneous bipartite structures and it controls for the transitivity similarity, avoiding the creation of unrealistic homogeneous degree distributions in the resulting unipartite graphs. We test this method using real world examples and compare the obtained results with alternative similarity measures, by validating the actual and orthogonal relations between the entities.

## Introduction

An object can be described by its attributes. Given a set of objects, it is often desirable to quantify the similarity between any two objects based on the attributes that they possess. A similarity measure is then used to predict the events in which these two objects behave similarly. For instance, one can ask whether two senators would vote concordantly given the similarity between their voting records. Or one can quantify the likelihood of a person switching occupations based on the task similarities between the occupations.

Here, we think of the object attribute associations as a bipartite graph of two types of nodes (i.e., objects and attributes), where a link is present (often with a weight) between an object and the attribute if the object possesses that attribute. Then, the object-object similarities can be modeled as a unipartite graph. Most of the recent interest in large-scale social, biological, and communication networks has been devoted to unipartite graphs [1], [2]. As a result, unipartite graphs are well understood in literature [3]. An impressive number of tools helps us extracting knowledge from such structures.

The methodology presented in this paper can be thought as a unipartite projection of an underlying bipartite graph. Many complex systems have an underlying bipartite representation: a scientist can be connected to papers that she wrote [7], an actor can be connected to a movie that he/she acted in [8], a country may be connected to the products it exports [9]; flavors can be connected to the food that they are tested in [10], human diseases can be connected to the genes that cause them [11] and many others (e.g., [4]–[6]). To exploit the richness of the methods developed for analysis of unipartite graphs in recent years, and, therefore, to gain an improved vantage point over the influence or interdependence of entities in bipartite structures, a unipartite projection becomes useful. For instance, projections of the bipartite graphs that we mentioned above has resulted in the scientific collaboration network [7], co-actorship network [8], the product space [9]; flavor network [10] or human disease network (diseasome) [11], respectively.

In network analysis, techniques aimed at uncovering the actual similarity value between entities in complex networks are popular. Some examples are the network back-boning technique to evaluate the significance of a link in weighted graphs [12]; or the graph deconvolution method to evaluate the direct connections between not directly connected entities [13]. Here, we are focusing on projecting bipartite graphs into unipartite entities. In the following, we refer to the graph projection as the construction of a unipartite graph map exploiting connections in a bipartite graph and allowing us to evaluate the similarity between the objects, for instance predicting which occupations are similar by looking at their common tasks.

**Figure 1:**
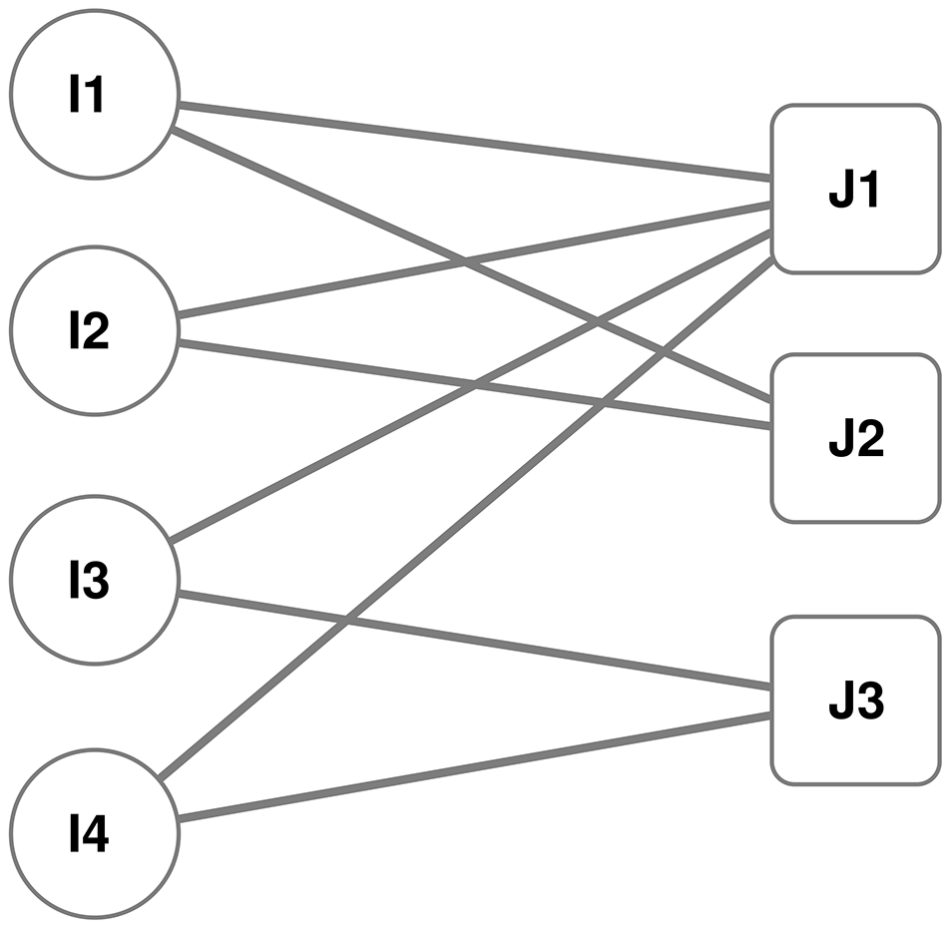
Toy example. This is a simple graph representation of a bipartite graph. Nodes in the class 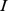 connect exclusively with nodes in the class 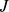 with 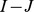 edges.

Projection techniques make use of distance measures and/or counting common elements [7], [14], [15]. The projection criteria are very important as they affect the usefulness of the graph itself. Suppose we want to create a network map of nodes of class 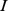 from Figure 1. Each node of class 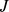 can be considered as a vectorial element. Then, the link strength in the bipartite graph would reflect the load of the class 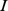 node in that dimension. Using this vectorial representation, then a classical spatial distance can be calculated, such as Euclidean or Cosine distance measures, which have been extensively used in adaptive filtering [16]. We can also represent a 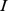 node as a set that contains all the 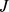 type nodes which it connects to. Then a set difference measure like the Jaccard measure can be calculated between all possible 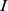 node pairings. Issues arise with these approaches. Simpler distance measures like the Jaccard measure cannot cope with what is known as the *saturation effect*: an additional shared 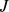 node between two 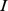 nodes that share only one 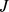 node should count more than between two nodes who already share 100 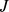 nodes [17]. Moreover, in a scale free bipartite graph, the degree of each node decays as 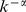 in both sets of nodes 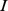 and 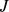. Therefore, few hubs from set 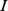 connect with many nodes in set 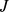, which on average have a low degree, as a consequence of the scaling of the power-law. If we project the structure to connect nodes from set 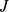, the low degree nodes will connect one with the other, as their few connections cannot outweigh their common 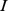 hub connection. Moreover, the similarity is transitive: 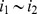 and 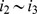 implies 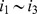. As a result, unipartite projections using these measures end up having a normal degree distribution, which is different than most real-world scenarios [18].

Some of these drawbacks do not affect other techniques. In [19] and subsequent papers by the same group [20], [21], authors propose to overcome the saturation problem by using a resource allocation process. In practice, each node in 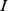 is considered to be a bearer of a certain quantity of resources, which it scatters equally to all its 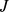 neighbors. Then, each node in 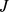 will disseminate equally all the resources it gathered back to 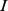 nodes. Using this process, we can quantify the information originated form an 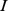 node and ended up in another 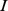 node. This amount is the degree of similarity of that node to the other 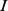 nodes. This approach, however, belittles crucial structural properties of the graph structure as a whole. The position of a node in the graph and the structural importance of the connections between 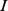 and 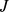 nodes influence their significance when projecting the graph, beyond what the simple degree can capture. In fact, the focus in [19] is to use bipartite graph projection as a tool for personal recommendation. In other words, the aim is not to predict an 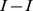 edge, but an 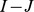 edge. In this case, the structural properties of the graph as a whole are not important, as the focus is in the direct neighborhood of the node. For example, in a customer-product bipartite graph where customers connect to the products they buy, the method presented in [19] aims to understand which products a customer will likely buy next, given what other customers similar to her purchased.

We have a different aim, namely to predict 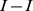 edges, that is equivalent of building the unipartite graph. In such scenario, we cannot just rely on the immediate neighbors but take into account the overall structure of the complex network. For this reason, we propose an approach that is alternative to [19], with a complementary application. In this method, we let a random walker explore the bipartite structure. In doing so, we can overcome most of the problems of traditional similarity measures. Two nodes from set 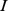 are similar if they frequently appear as successive visiting sites of the random walker. Since hubs in 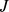 are connected to many nodes in 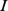, their contribution to each node pair in 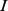 is low, as the probability of consistently choosing the same endpoints can be considered insignificant. In this way, the random walker gives us information taking into account the overall structural properties of the graph. Random walkers have been extensively used in literature with this precise aim. For example, they are at the basis of the modular organization detection of many community discovery algorithms [22], [23]. Other applications include centrality measures, used to rank nodes according to their structural importance [24].

The numerical simulations indicate that this approach is able to predict 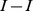 edges with higher confidence, when the unipartite graph maps extracted from the bipartite graphs are tested against the real world knowledge about the 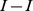 connections. This happens in four different realms, including occupation flows, industry employee flows, political activities in the US Congress and a citation graph between international aid agencies. We also tested our method in ranking 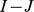 edges and it functions equally well as the other methods.

## Methods

The proposed approach consists of projecting the bipartite graph into a unipartite graph by creating a weighted edge between two nodes in the unipartite graph from the information present in the bipartite graph. The weight is directly proportional to how often one would observe a random walker on the bipartite graph visiting the two nodes consequently. Formally, let us assume that in the bipartite graph there are two types nodes indexed with 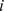 and 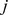, respectively. Assume that there are 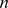 (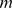) 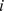 (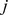) type nodes that form the set 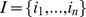 (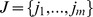). An edge in the bipartite graph is defined as a link between an 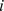 type and 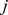 type node. We can define the 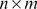 adjacency matrix, 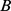, whose 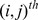 entry represents the strength of the links between node 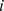 and node 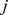. In the binary case, 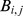 will be 1 if there is an edge between 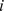 and 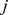, and 0 otherwise. In general, a bipartite graph can be represented by the triplet 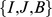. The unipartite projection of this bipartite graph onto 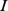 domain requires defining an 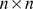 edge matrix, 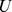, from the bipartite edge matrix 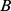.

Here, we propose to build the 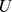 matrix as the number of times a random walker (RW) passes through a pair of 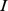 type nodes on the bipartite graph, separated by a single 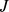 type node. Suppose the RW is on the node 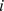. Then, the RW would end up to any 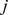 type node with probability:
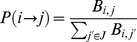
(1)


Once on a 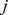 type node, the probability that the RW goes to node the 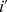 is:
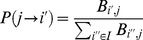
(2)


Therefore, the probability of moving between nodes 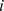 and 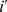 will be the sum of all paths 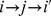 that pass through 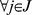:
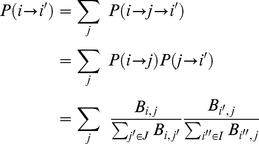
(3)


We can rewrite the transition probabilities in terms of a Markov transition matrix 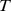, such that 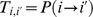. The frequency of observing the path 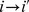 also depends on how often the RW visits node 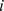 in general. Suppose that 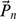 denotes the probability vector whose 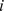
th element is the probability of the RW being on the node 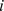 in the 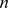
th step of the random walk. We initialize the process with 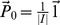 where 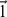 denotes a row vector of ones. Therefore:
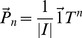
(4)


Since 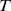 is a right-stochastic matrix (i.e., its elements are non-negative and sum of its rows is always 1), the stationary distribution, 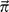 will satisfy:
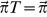
(5)


Here, we will assume that the transition matrix is irreducible (i.e., every node can communicate with each other in finite step) and aperiodic (i.e., there is no 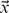 and integer 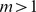 such that 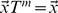 but 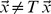). If any of these properties are violated, then we will not be able to ensure a unique stationary distribution. In our case, we would ensure that the bipartite graph is connected, which would satisfy the irreducibility property. Moreover, we only work with bipartite graphs with non-directed edges which justifies the aperiodicity property. With these properties at hand, the Perron-Frobenius theorem guarantees the existence of unique stationary distribution, which is the left eigenvector of 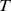 matrix with eigenvalue 1.

Given that we calculated the stationary distribution 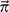, then as the RW moves infinitely many times, the random-walk similarity between nodes 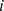 and 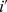 is:
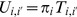
(6)


We would like to remark that Zhou et al. [19] defines a similar metric based on their ProbS methodology but they they do not include 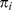 in their definition. The 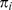 element is the one that contributes information about the overall graph structure. It allows the similarity to consider not only the immediate neighbors of a node, but also its position in the graph, enabling 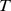 to avoid the saturation and transitivity issues described in the Introduction.

### Other Projection Techniques

In this section we provide our implementation of the methods we compared our technique with. In each technique, the entities of the bipartite graph 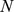 are considered as binary vectors. Suppose that we have a bipartite graph with two classes of entities 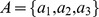 and 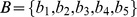. Suppose that 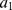 is connected to 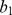, 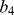 and 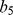. Then 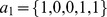. In the following discussion, we adopt the convention of always projecting onto nodes of class 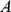.

#### ProbS

This is the bipartite projecting technique presented in [19]. The assumption at the basis of this measure is the same we implemented, namely that the relatedness of 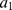 and 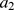 depend on the resource flow from 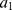 and 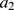 to the 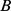 nodes and back. Instead of implementing this idea with random walks, Zhou et al. decided to pass the entire resource equally to all 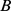 nodes and back.

So, in the first step, all the resource flows from 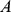 to 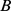 as:
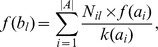
where 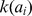 is the degree of 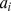 and 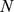 is the 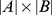 adjacency matrix representing the bipartite graph, containing 1 if 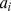 is connected to 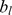, 0 otherwise. In the next step, all the resource flows back to 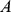, and the final resource located on 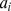 reads:



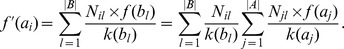



This can be rewritten as:
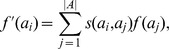
where:



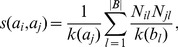
(7)which sums the contribution from all two-step paths between 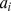 and 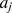, and it is ultimately the similarity between the two nodes.

Using a standard example that will be adopt from now on, we assume that 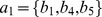 and 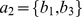, and all 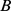 nodes do not have any other connection with any other 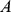 node. Then, 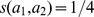.

#### HeatS

Heats method, introduced by Zhou et al. in [20], is related to the ProbS method but instead of normalizing by column, it is normalized by the row. Mathematically,
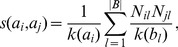
(8)


The difference between HeatS (Eq. 8) and ProbS similarity measures (Eq. 7) is the first fraction. For the example introduced above, HeatS similarity would be 1/6, lower than ProbS similarity of 1/4.

#### Hybrid

The Hybrid methodology, introduced in [21], hybridized ProbS and HeatS, by taking a geometric average of the first normalizing parameters. The similarity in this measure is defined as:
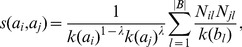
(9)


Assuming 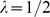, the similarity will be 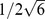, a value between ProbS and HeatS similarities.

#### Jaccard

In this bipartite projecting technique, each class 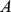 node is seen as a set of elements. So, if 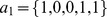, then we consider it equivalent to 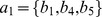. Then, the similarity between two nodes 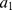 and 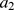 is equivalent to the Jaccard similarity:
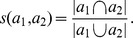



For instance, if 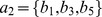, then 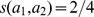.

#### Cosine

This bipartite projecting technique is based on the Cosine similarity. The Cosine distance between two vectors of same length is defined as:
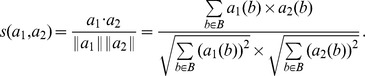



We recall that *a*_1_ and *a*_2_ are both binary vectors. For each 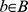 where either (or both) nodes are not attached, the overall contribution to the sum is 0. Only when both 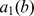 and 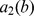 are equal to 1 there is a contribution of 1 to the sum.

Again considering our standard example 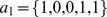 and 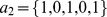, we obtain 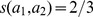.

#### Euclidean

The Euclidean projecting technique takes advantage of the concept of Euclidean distance. The *a*_1_ and *a*_2_ vectors are seen as points in a 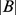-dimensional space. We then calculate the Euclidean distance between points *a*_1_ and *a*_2_ as follows:
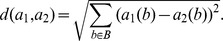



The Euclidean similarity is inversely proportional to the Euclidean distance, thus 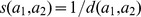. Euclidean similarity gives more weight not only to the co-presence of 1 s in *a*_1_ and *a*_2_, but also in co-presence of 0 s.

Keeping fixed 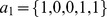 and 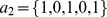, we obtain 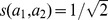.

#### Pearson

This is the bipartite projecting technique based on the well-known Pearson correlation coefficient. We calculate the correlation coefficient of *a*_1_ and *a*_2_ vectors as follows:
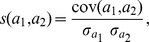
where 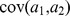 is the covariance of *a*_1_ and *a*_2_, and 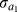 and 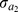 are the variance of the *a*_1_ and *a*_2_ vectors, respectively. Just like in the Euclidean case, also the Pearson similarity gives some weight to the co-absence of a connection, not only a co-presence.

We calculate the Pearson similarity for our standard example in which 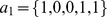 and 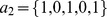, obtaining as result 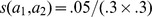.

## Results

We create two different sets for our experiments. In the first one, we compare the performances of all the methods using the experiments described in [19], on the very same dataset extracted from MovieLens and processed as described there. We then refer to [19] for the details of this experiment. Aim of the first experiment is to test the efficiency of different methods in ranking 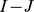 edges. In a second set of experiments, we study the projection of four real-world bipartite graphs. In this case, we also have unipartite graphs with observed relations between *I* entities. Aim of this experiment is to show that the 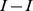 edges, as ranked by the proposed methodology, are closer to the observed relations than any other methodology.

In both experiments, we compare the obtained results with the seven alternative projecting techniques presented in the previous section. Four of them are based on distance measures: Jaccard, Cosine, Euclidean and Pearson. The other three alternatives are ProBs [19], HeatS [25] and Hybrid [20]. We refer to the proposed method as Bipartite Projection via Random-walk, or “BPR”.

### I-J Edges

In this numerical simulation, we have user-to-movie connections if a user (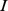) liked the movie (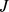) and the aim is to suggest other movies to the user (a 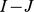 edge). To test the efficiency of the methods, a random subset of connections are removed from the original bipartite graph. Then we calculate movie to movie similarity in the remaining graph using the measures presented above. Finally, for each user 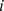 and movie 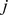 we average (for Cosine, Euclidean, Jaccard and Pearson) or sum (for ProbS, HeatS, Hybrid and BPR) the movie similarities to 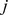 of all movies which are liked by 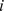. At the end of the procedure, for each user 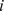 we have a list of 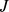 nodes, sorted by the computed value. We calculate the quality of this suggestion list in two ways. First, given a user 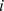 and a movie 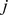 that was removed from the graph, 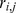 is equal to the rank of 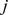 in 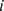
's suggested list over the length of the list. Second, we shorten the suggested list to different lengths (including 10, 20 and 50 elements) and we record the share of the randomly removed movies that are included in the list - we refer to this measure as Hit Rate (HR-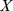, where 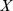 is the length of the recommendation list). Hybrid method also includes a parameter of choice (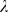 in Equation 9). We selected 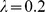, which maximized the predictive power.

**Figure 2:**
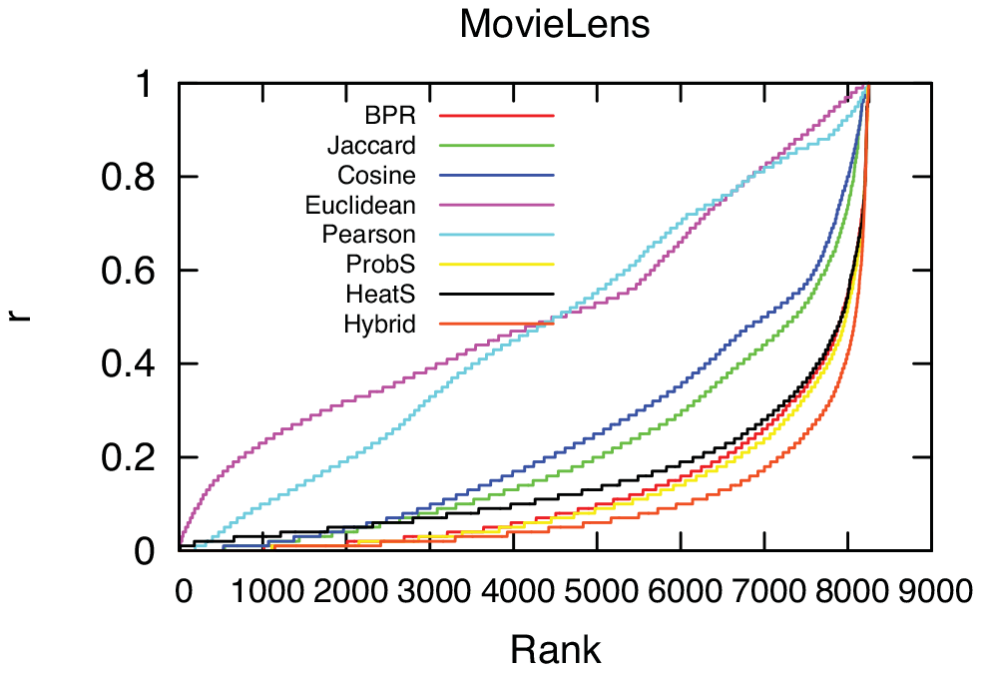
The predicted position of each entry in the probe ranked in the ascending order. On the y-axis, 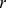 measures the position of an 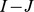 edge (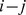) in the ordered result of the prediction. For example, if there are 1500 uncollected 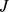 connections for 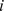, and 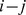 is the 30th strongest prediction, we say the position of 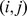 is the top 30/1500, denoted by 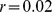. Lower 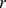 values indicate better predictions.

**Figure 3:**
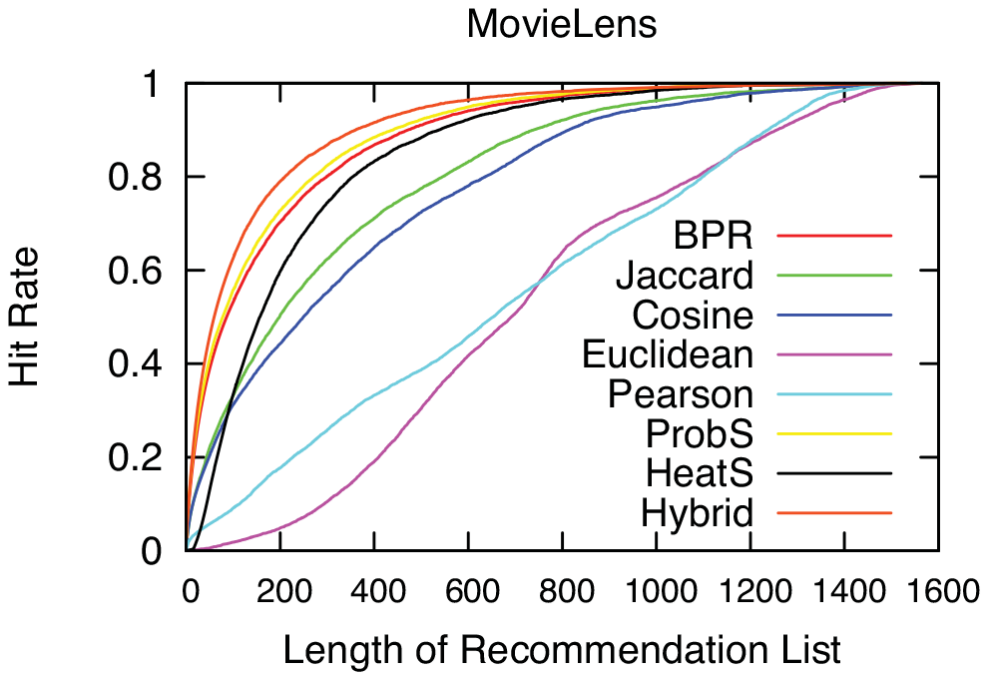
The hitting rate as a function of the length of recommendation list. We shorten the recommendation list to increasing values, in the x-axis, and we report the share of actual 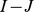 edges predicted, on the y-axis. The higher the hit rate, the better the prediction.

The results of this numerical simulation are reported in Figures 2 and 3, and Table 1. In Figure 2 we report the cumulative value of 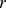 as the recommendation lists grows. A lower value here indicates a better prediction method. In Figure 2, ProbS, Hybrid, BPR and HeatS appear to easily outperform all other methods, with Hybrid performing the best. BPR performed better than HeatS but was slightly worse than ProbS. In the first column of Table 1 we report the overall average value of 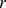. The ranking of the methodologies remains the same.

**Table 1:** Average predicted position (<*r*>) and hit rate for recommendation lists of length 10 (HR-10), 20 (HR-20) and 50 (HR-50).

Distance	<*r*>	HR-10	HR-20	HR-50
BPR	0.12336	14.65%	23.02%	38.68%
ProbS	0.11417	15.97%	24.57%	40.99%
Jaccard	0.21086	7.97%	12.30%	22.05%
Cosine	0.24151	7.87%	12.16%	20.35%
Euclidean	0.50581	0.06%	0.16%	0.53%
Pearson	0.46280	2.62%	3.59%	5.68%
HeatS	0.15221	0.21%	1.55%	13.28%
Hybrid	0.08814	17.94%	27.61%	46.08%

In Figure 3 we report the hit rate at different lengths of the recommendation list. Again, the result is confirmed: Hybrid, ProbS and BPR outperform in the task with respective order. The hit rates at different list length are reported in the HR-X columns of Table 1. All these results confirm that BPR works in 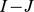 but there are more efficient methodologies, namely Hybrid and ProbS in this task.

### I-I Edges

For the task of predicting 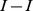 edges we consider four different bipartite graphs:

- Occupations connected to the tasks they fulfil, from the O-Net database [26],[27] (referred here as “O-Net”).
- Industries connected to the fields of educations of the people they employ, from the IPUMS dataset [28] (referred here as “IPUMS”).
- International aid organizations connected to the countries and the development issues they talk about in their websites [29] (referred here as “Aid”).
- Congressmen from the 111th US Congress, connected to the topics they wrote a bill on (referred here as “Congress”).

For additional information about how we built the bipartite and the unipartite graphs used, see Material S1.

For each of this bipartite graph we have a corresponding unipartite graph that we use to evaluate the goodness of the projection. The test graphs are:

- For O-Net dataset, the occupation-occupation job flows.
- For IPUMS dataset, the job flows across industries.
- For Aid dataset, the mentions of other aid organizations in an organization's website.
- For Congress dataset, the co-sponsorship of bills.

**Figure 4:**
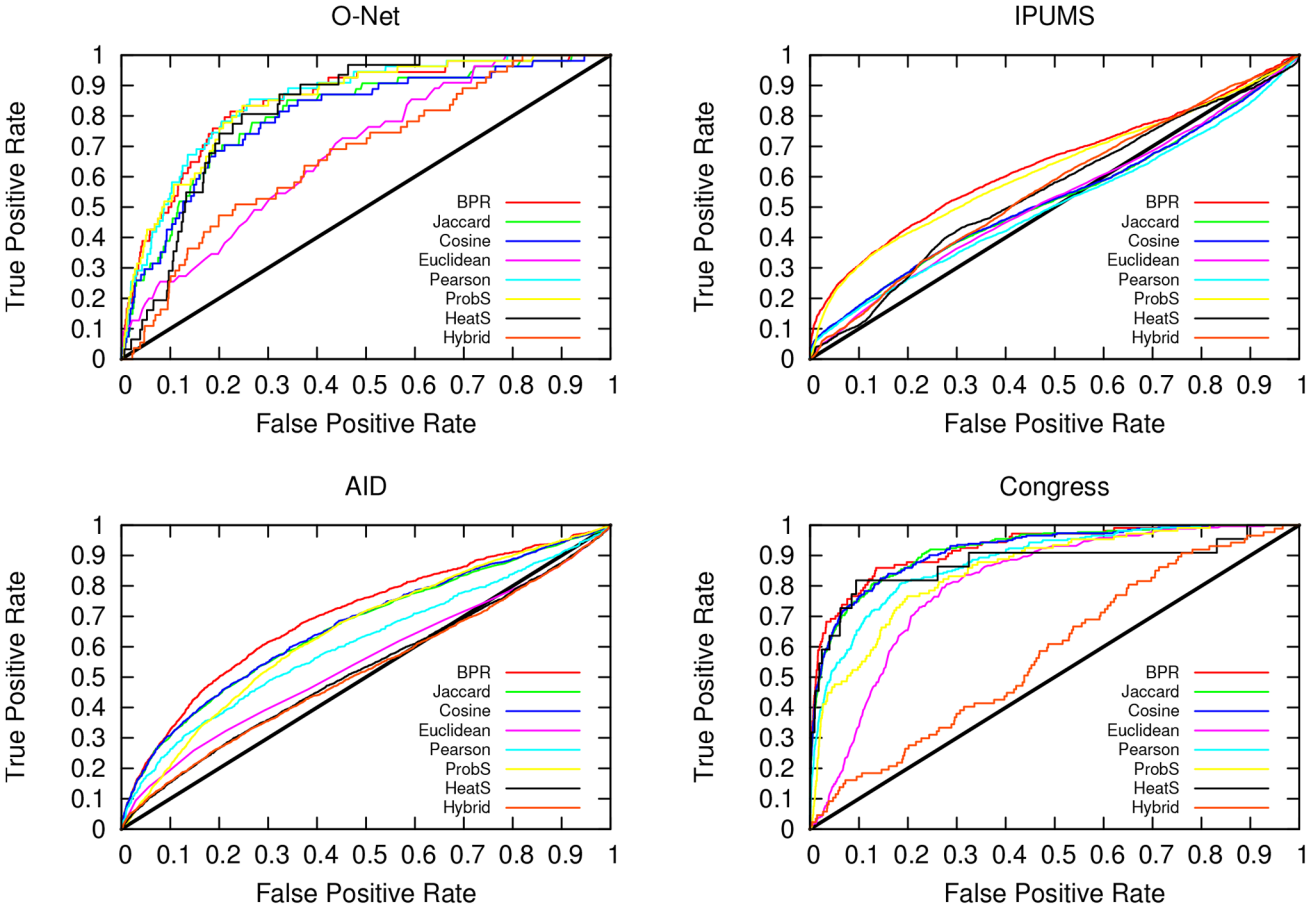
ROC Curves. The receiver operating characteristic (ROC) curves for the four datasets in our experimental set up: O-Net (top left), IPUMS (top right), Aid (bottom left) and Congress (bottom right). Each predicted 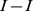 edge is sorted according to the prediction confidence and it is tested against the observed real-world graph.

The procedure is the same presented in the previous section: for each pair of 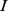 nodes we calculate the similarity using one of the proposed measures. For each node 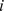, we obtain a ordered list of similarities. We use this list to predict actual 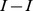 edges, observed in the corresponding unipartite graphs. In Figure 4 and Table 2 we report the performance in the prediction task for all methods and for all graphs. Figure 4 presents the receiver operating characteristic (ROC) curves of the various methods. We can see that BPR comes as winner or a close second in most cases. Table 2 reports the area under the ROC curve, that summarizes the overall quality of the predictions shown Figure 4. Table 2 confirms that BPR is the best predictor of the 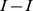 edges, based on the observed 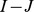 edges in the test graphs, with the exception of the O-Net graph. However, in that case, BPR is beaten by Pearson, which scores poorly in the other scenarios. The second best predictor is different for each graph, while BPR's performance across all graphs is constantly on top. Since we are dealing with the weighted graphs, we need a threshold, 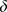, to determine when an observed weight is significant and when it is not. 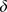 influences prediction scores, but not the performance ranking of the methods (see Material S1 and Figure S1).

**Table 2:** AUC Values.

Distance	O-Net	IPUMS	Aid	Congress
BPR	0.84627	**0.62831**	**0.69724**	**0.93063**
ProbS	0.84528	0.61966	0.64224	0.90438
Jaccard	0.80507	0.52031	0.66255	0.91908
Cosine	0.79768	0.52101	0.65484	0.91806
Euclidean	0.68452	0.50441	0.53616	0.80787
Pearson	**0.85186**	0.50380	0.65881	0.85145
HeatS	0.78843	0.53862	0.52121	0.88540
Hybrid	0.67216	0.56061	0.51862	0.57986

The AUC is the integral of the area below the ROC curve, as shown in Figure 4. If we obtain an AUC equal to.5, then the prediction is said to have a performance equivalent to a random predictor.

**Figure 5:**
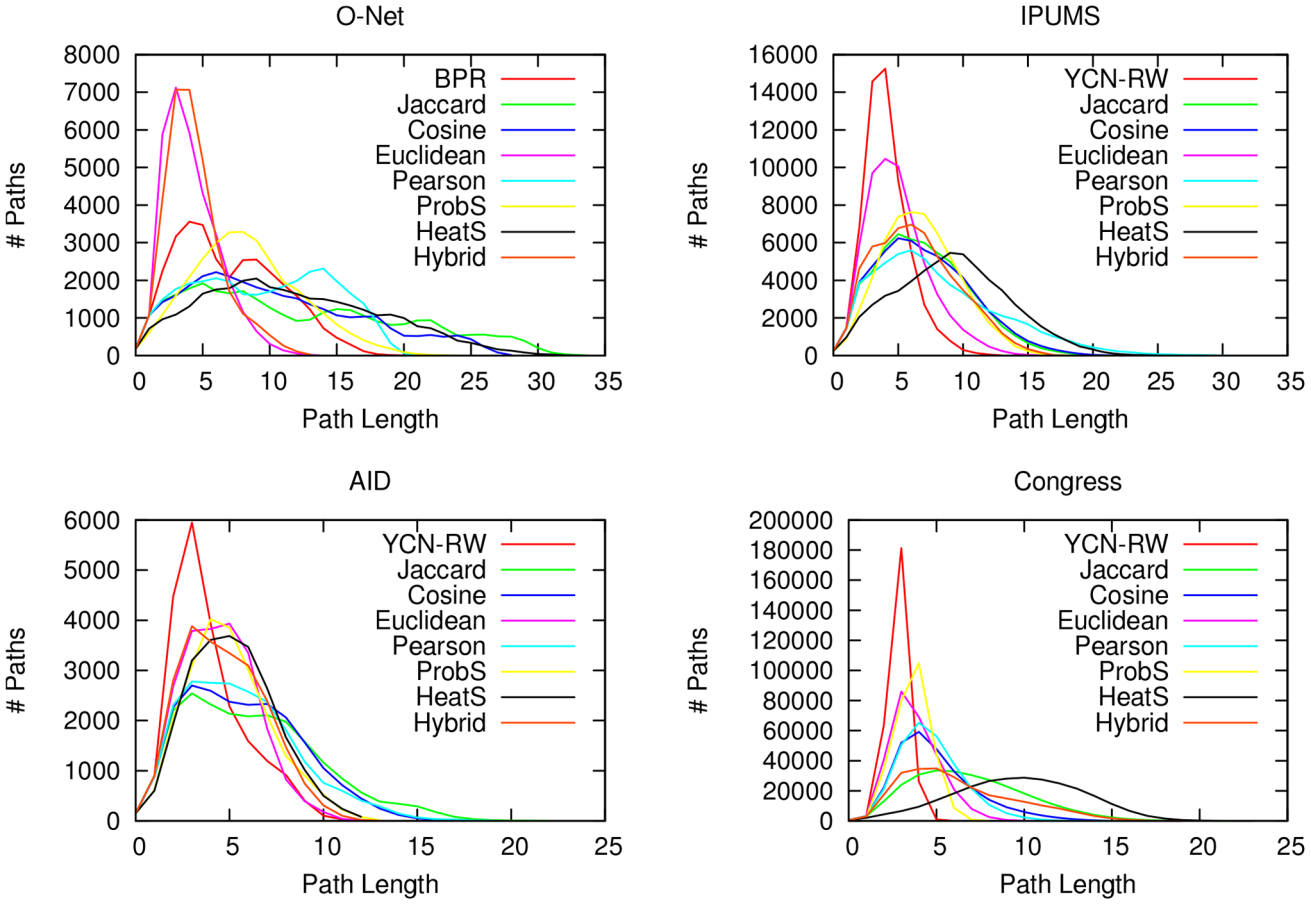
Path distributions. The distribution of the shortest paths in the unipartite graphs generated with each technique, for all datasets: O-Net (top left), IPUMS (top right), Aid (bottom left) and Congress (bottom right). We count the number of paths (y-axis) with a given length in number of edges (x-axis).

Prediction quality is not the only quality criterion to evaluate the unipartite projections. We also want the unipartite graph map to have topological properties comparable to the real-world complex graphs in the literature. One of such properties is the small-world property [30]: the distribution of shortest paths are normally distributed around a mean much lower than the random Erdös-Renyi graphs, usually 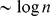 where 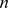 is the number of nodes in the graph [3]. Figure 5 shows the distribution of the shortest path lengths in different bipartite graph projections. Each graph map has been generated by extracting the maximum spanning tree from all the 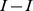 edge similarities returned by each method, and then adding edges until the average degree reaches 3. We can see that BPR is the only method which constantly generates unipartite graphs with the expected distribution of shortest path lengths. With the exception of the Euclidean method in the O-Net dataset, the other methods have usually either higher averages or distributions more skewed on higher values, or both.

**Figure 6:**
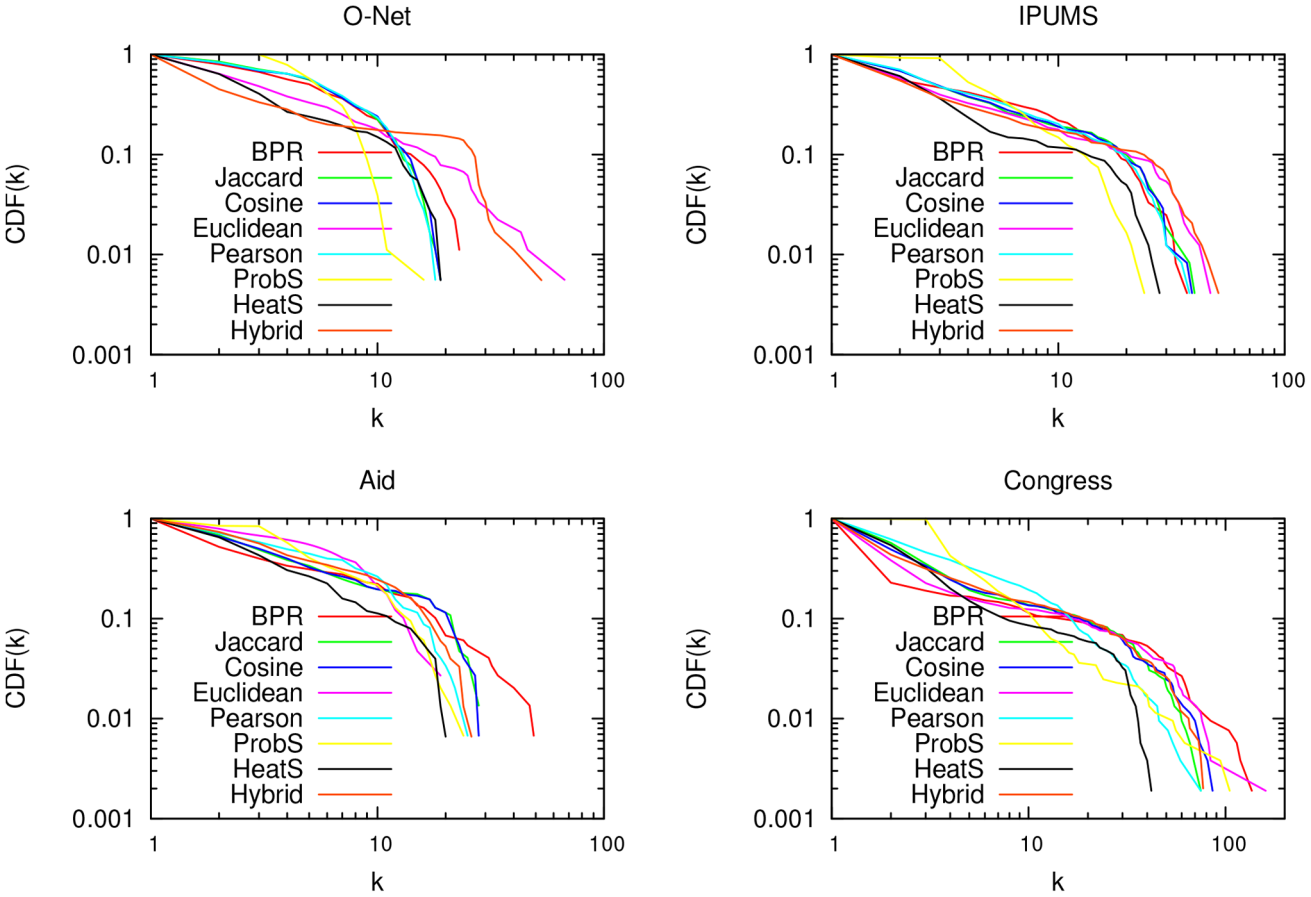
Degree distributions. The cumulative degree distributions of the unipartite graphs generated with each technique, for all datasets: O-Net (top left), IPUMS (top right), Aid (bottom left) and Congress (bottom right). We calculate the probability (y-axis) for a node to have a degree equal to or higher than a given degree (x-axis).

Another property of real-world graphs is a broad degree distribution. Real-world graphs are characterized by few hubs with high degree and many nodes with degree equal to one [18]. However, transitive similarity measures may be prone to boost transitivity beyond what is reasonable, creating large cliques and inflating the degree of most nodes. Therefore, for a similarity measure, higher skewed distribution is a desirable property because it is a signal of the absence of large cliques, that lowers the practicality of the network map. We depict the cumulative degree distributions of the graph projections in Figure 6. We can see that BPR has very broad degree distributions, clearly the broadest in the Aid graph and the broadest in Congress and O-Net after the Euclidean graph. However, we saw that for practical purposes the only contestant was ProbS (Figure 4 and Table 2). Here, ProbS is affected exactly by the problems of very homogeneous degree distribution: in all graphs the nodes with degree lower than 3 are very few (always less than 10%), while the most connected nodes have half or a third the degree they have in BPR.

## Discussion

The proposed bipartite projection technique gives differential weights to elements by their commonality. The methodology generates an edge in the graph map whenever the random walker frequently visit the two nodes in the same path, traversing their common elements, which ensures that hubs do not artificially drive up the similarity measure. As a result, the random walk similarity allows the creation of significant and meaningful graph maps, who are structurally very similar to the corresponding real-world graphs. Consequently, the resulting graph projections carry some fundamental properties that are observed in many other naturally occurring graphs.

As a criticism, one could say that it only works in the case of bipartite graphs that exhibits non-overlapping scale free degree distributions, where there are hubs in one or all classes of nodes. In any case, any projecting technique has limitations, and the choice between one algorithm over another has to be made considering the objective of the exercise. We do not exclude the existence of a scenario in which our methodology will not yield significant results. Yet, it has been proved that broad (scale free or exponential) degree distributions are ubiquitous in real world graphs: from social graphs to scientific co-authorship, from the physical Internet infrastructure to the virtual hyperlinks in the World Wide Web, from financial graphs to protein interactions. Therefore, we conclude that our methodology may be applied in this wide range of scenarios.

## Supporting Information

Figure S1
**Threshold sensitivity.** AUC values for different threshold (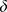) choices in four datasets: O-Net (top left), IPUMS (top right), Aid (bottom left) and Congress (bottom right). See Material S1 for details.(EPS)

Material S1 (PDF)
