# Supplementary material for: Using Random Walks to Generate Associations between Objects
Source: PLoS One. 2014 Aug 25;9(8):e104813. doi: 10.1371/journal.pone.0104813 (PMC4143196; doi:10.1371/journal.pone.0104813)
Supplement: Material S1 — (PDF) [file pone.0104813.s002.pdf]

Supplementary Material

Muhammed A. Yildirim[1,*] Michele Coscia[1]
1 Center for International Development, Harvard University, 79 JFK St, Cambridge MA, US 02138
∗ **E-mail: Corresponding muhammed_yildirim@hks.harvard.edu**

Data Collection and Selection

In the paper we make use of four datasets to evaluate the quality of our projections. Each dataset has to be composed by two parts. The first part, N, is the bipartite network that needs to be projected. The second part, U, is a unipartite weighted network composed by all elements of one class of entities of the bipartite network. The unipartite network has to be an observation of a direct connection between these entities, it cannot be created with a different projection of another bipartite network.

For the O-Net and the IPUMS datasets, the unipartite networks come from an external data source, namely the Current Population Survey (see the Subsection CPS). For the Aid and the Congress datasets, the unipartite network is part of the original dataset itself.

Data and code is publicly available at `http://www.michelecoscia.com/?page_id=734`.

O-Net

The O-Net dataset refers to data downloaded from the O-Net platform (`http://www.onetcenter.org/`). The O-Net program provides comprehensive occupational descriptions and data for use by job seekers, workforce development offices, human resources professionals, students, researchers, and others. O-Net identifies the knowledge, skills, abilities, activities and tasks that are distinguishing characteristics of an occupation. The O-Net Content Model defines the key features of an occupation as a standardized, measurable set of 277 variables. The included 974 occupations are organized using a proprietary variation of the 2010 SOC taxonomy, called O-NET-SOC.

O-Net data can be viewed as a bipartite network connecting knowledge, skills, abilities, activities and tasks to occupations. Here, we focus on a view centered on occupations, i.e. we consider only the connections between occupations and the other five classes of entities. Task and activities are associated to occupations without weights, i.e. either they are connected or not.

Knowledge, skills and abilities are instead connected with all occupations with a given weight, a continuous value in the range $[0, 7]$ that represent the relevance for that occupation. We decide to binarize these connections in the following way. For each knowledge, skill and ability code c we create three derived codes c_{low}, c_{med} and c_{hi}, representing a low, medium and high level of relevance for the occupation. Then, we establish three thresholds in such a way that, for each c, 50% of occupations are connected to c_{low}; 12.5% of occupations are also connected to c_{med} and 2.5% of occupations are also connected to c_{hi}.

We recursively remove occupations with degree lower than 4 and knowledge, skills, abilities, activities and tasks with degree lower than 2. We end up with 428 occupations and $3,929$ nodes in the other five categories. The total number of connections in N_{O-Net} is $69,013$.

IPUMS

The Integrated Public Use Microdata Series (IPUMS-USA) (`https://usa.ipums.org/usa/index.shtml`) consists of more than fifty high-precision samples of the American population drawn from fifteen federal censuses and from the American Community Surveys of 2000-2011 [1]. From the official website, we downloaded the occupation and industry variables for each individual included in the surveys of 2009, 2010 and 2011. The industries are classified using the NAICS classification system.

We counted the number of times an industry and an occupation are connected ($x_{i,o}$). We also counted the total amount of people in a given occupation ($x_{*,o}$) as well as in a given industry ($x_{i,*}$). We created a bipartite industry-occupation network connecting an industry i to an occupation o if:

$$\frac{x_{i,o}}{x_{i,*}} \Big/ \frac{x_{*,o}}{x_{*,*}} > 1,$$

where $x_{*,*}$ is the total number of people included in the surveys. In other words, we connected an industry to an occupation if the number of people in that occupation exceeds the fair share that the industry would get, given its size, if occupation would distribute at random among all industries.

We recursively removed all industries and occupations that have degree lower than 1. We end up with 267 industries and 513 occupations. The final number of connections in the bipartite graph N_{IPUMS} is $18,104$.

CPS

From the O-Net dataset we want to obtain a unipartite occupation-occupation projection; from the IPUMS dataset we want to obtain a unipartite industry-industry projection. To create the corresponding unipartite networks U_{O-Net} and U_{IPUMS} we need an independent data source containing a measure of similarity between these entities.

We downloaded data between January 2003 and December 2010 from Current Population Survey (CPS) (`http://www.census.gov/cps/`). The CPS is administered by the Census Bureau using a probability selected sample of about $60,000$ occupied households. Households are in the survey for 4 consecutive months, out for 8, and then return for another 4 months before leaving the sample permanently. We selected all subjects who are employed and we extracted their occupations and industries from both the 4-month windows in which they are present. We connect an occupation (industry) to another occupation (industry) if the subject switched between the two.

In the CPS, industries are classified using the NAICS codes, therefore the correspondence with IPUMS data is one-to-one. The weights of the edges of U_{IPUMS} are simply the number of people switching from one industry to the other. Occupations are classified using the original 2010 SOC classification, thus it is vastly overlapping with the O-Net classification. When there is a one-to-one correspondence between the codes, the weights of the edges of U_{O-Net} are the number of people switching from one occupation to the other. However, in some cases to one SOC code there are multiple O-NET-SOC codes. In these cases the weights of the edges of U_{O-Net} are the number of people switching from one occupation to the other divided by the number of matching O-NET codes.

U_{O-Net} contains $20,822$ weighted edges, while U_{IPUMS} contains $37,938$ weighted edges.

Aid

The Aid dataset (`http://www.atlas.cid.harvard.edu/aidxp/`) corresponds to the dataset used in [2]. It has been constructed by creating a custom search engine in Google indexing the websites of the largest 152 international aid organizations. The custom search engine has been systematically queried to evaluate the number of web documents containing the name of an aid organization and an issue, the name of a aid organization and a country, and an issue and a country.

The countries considered are the 110 with lowest GDP per capita and at least one million inhabitants. The list of issues includes 34 development issues such as school completion and HIV/AIDS. For more information about the creation of the dataset, we refer to the original paper.

Our aim is to evaluate the similarity between two aid agencies. To this end, we select as N_{Aid} the tripartite network centered on aid organizations, considering their connections to countries and to issues. In this network, each edge is weighted with the number of hits containing both the aid organization and

the country (issue). We keep only the edges with a weight equal to at least 100, resulting in a total number of $13,612$ edges for N_{Aid}.

The original dataset provides also the organization-organization unipartite network U_{Aid}. Also in this case, each edge is weighted with the number of hits. The total number of edges is $13,161$.

Congress

The Congress dataset has been downloaded from the web (`http://www.govtrack.us/`). Govtrack is a website that records all actions of the US congress for each bill, whether it is enacted and signed or not. We downloaded all the bills from the 111th US Congress. Each bill is connected with a list of sponsors (the members of the Congress who signed it) and a list of subjects.

For the $N_{Congress}$ network, we connect each member of the Congress with the list of topics for which he signed at least one bill. $N_{Congress}$ is a bipartite network connecting the 525 members of Congress to 618 topics (that are shared by at least two congressmen) with $56,215$ links. For the $U_{Congress}$ network, we connect each member of the congress with a weighted edge proportional to the number of bills they co-sponsored. We end up with $106,410$ weighted edges.

The Significance Threshold for the Test Networks

The U unipartite networks we use to test how good is the projection of the network N are weighted, a necessary requirement to calculate the ROC curves of Fig. 2 and the AUC values of Tab. 1 in the paper. Given heterogeneous weight distribution, we come up with a threshold δ, to divide the weights that are significant from the ones that are not. We report in Tab. 1 the values of δ used in the paper.

The meaning of δ in different U networks is the following:

- **O-Net**. Here the U network represents transitions from an occupation to another: δ is the number of transitions required between the two occupations to keep the edge.
- **IPUMS**. Here the U network represents transitions from an industry to another: δ is the number of transitions required between the two industries to keep the edge.
- **Aid**. Here the U network represents the number of mutual mentions that the aid organizations made through their websites: δ is the number of mentions required to keep the edge.
- **Congress**. Here the U network represents the bill co-authorship of congressmen: δ is the number of bills, co-authored by the two congressmen, required to keep the edge.

However, if we use different thresholds we are changing the final results, as some existent weak edges are excluded and some are included for different values of the threshold δ. A lower value of δ allows more noise to pass through the filter. Higher values of δ filter too much information, resulting in less “actual” edges. Intuitively, the AUC values tend to decrease as δ decreases (more noise implies a more difficult prediction) and to increase as δ increases (less and stronger edges are easier to predict), although there are exceptions.

To guarantee the robustness of the results published in the paper, we depict in Fig. 1 the AUC values for different thresholds. In Fig. 1, the vertical black line is placed on our choice of δ, and the corresponding AUC values are the one reported in Tab. 1 in the main paper.

We can see that in all datasets where our technique had a significant edge (O-Net and IPUMS), the choice of δ does not really make a significant difference. The distance in performance is more or less constant, with the exception of the Euclidean distance in the IPUMS network, but only for growing values of δ. For the networks where our method shows just a marginal improvement, we can see that it either sits comfortably on top for all threshold values (Aid network) or it has not a significant difference with all the other methods and it comes up first for most values of δ (Congress).

References

1. Ruggles S, Alexander JT, Genadek K, Goeken R, Schroeder MB, et al. (2010) Integrated public use microdata series, ver. 5.0 (machine-readable database). Minneapolis: University of Minnesota .

2. Coscia M, Hausmann R, Hidalgo CA (2013) The structure and dynamics of international development assistance. Journal of Globalization and Development : 1–42.

3. Zhou T, Ren J, Medo M, Zhang YC (2007) Bipartite network projection and personal recommendation. Physical Review E 76: 046115.

Tables

U	δ
O-Net	140
IPUMS	35
Aid	12
Congress	7

Table 1. The value of δ for each network U.
